# Life-Threatening Secondary Adrenal Crisis Triggered by Pembrolizumab-Induced Hypophysitis: A Case Report

**DOI:** 10.7759/cureus.111692

**Published:** 2026-06-28

**Authors:** Aye Nyein Thu, Munir Babar

**Affiliations:** 1 Diabetes and Endocrinology, Worcestershire Acute Hospitals NHS Trust, Worcester, GBR

**Keywords:** autoimmune hypophysitis, hypogonadotrophic hypogonadism, hypopituitarism, immune checkpoint inhibitors (icis), immune-related adverse events (iraes), melanoma, pd-1 inhibitor, pembrolizumab, pembrolizumab-induced hypophysitis, secondary adrenal insufficiency

## Abstract

The utilization of immunotherapy and targeted therapies in advanced malignancies is rapidly increasing due to advancing technologies. While highly effective, the unintended toxicities and immune-related adverse events (irAEs) associated with these treatments require vigilant monitoring. Autoimmune hypophysitis and subsequent pituitary dysfunction represent critical complications of immune checkpoint inhibitors (ICIs), particularly pembrolizumab. Hypopituitarism can manifest as panhypopituitarism leading to a life-threatening secondary adrenal crisis or as selective anterior pituitary hormone deficiencies, such as isolated adrenocorticotropic hormone (ACTH) or gonadotropin deficiency. We report the case of a 73-year-old man with recurrent melanoma who presented with pituitary dysfunction secondary to pembrolizumab therapy. This case highlights the necessity of investigating, screening, and promptly treating new constitutional symptoms in patients undergoing ICI therapy, as these endocrine complications can be fatal if left unrecognized.

## Introduction

The landscape of advanced malignancy management has been revolutionized by the advent of immune checkpoint inhibitors (ICIs), including pembrolizumab and nivolumab [1]. These agents are primarily deployed in solid malignancies such as melanoma and colorectal, endometrial, and lung cancers [1]. ICIs function by targeting various pathways, including programmed death receptor-1 (PD-1), programmed death-ligand 1 (PD-L1), and cytotoxic T-lymphocyte antigen 4 (CTLA-4) [1]. Pembrolizumab, a PD-1 inhibitor, blocks the PD-1 protein on T cells, effectively disinhibiting the immune system and restoring anti-tumor activity [1,2].

Although ICIs, including pembrolizumab, can induce durable remissions in end-stage metastatic disease, they are frequently associated with immune-related adverse events (irAEs) resulting from generalized immune system hyperactivation [1]. These irAEs can affect diverse organ systems, leading to colitis, pneumonitis, hepatitis, myocarditis, and widespread endocrinopathies such as thyroiditis, adrenalitis, and hypophysitis [1]. Immune-related hypophysitis is a rare but critical complication that demands heightened clinical awareness [2]. This report details the case of a patient who developed secondary adrenal insufficiency following pembrolizumab therapy for facial melanoma, emphasizing the importance of recognizing insidious hypopituitarism symptoms in oncological patients.

## Case presentation

Initial investigations

A 73-year-old man presented to the Accident and Emergency (A&E) department reporting profound fatigue, generalized malaise, anorexia, lightheadedness, and drastically reduced exercise tolerance. He was urgently referred by an out-of-hours general practitioner after noticing his random serum cortisol level returned alarmingly low at <11 nmol/L. The patient noted these symptoms had been progressively worsening over the preceding three to four months. Upon further systematic inquiry, he reported a recent reduction in shaving frequency, loss of libido, and erectile dysfunction, which he had not previously reported.

The patient had a history of recurrent superficial spreading melanoma on his right cheek. Six months prior to admission, he was initiated on adjuvant pembrolizumab (400 mg every six weeks) following surgical excision. Therapy was discontinued two months prior to presentation (after three cycles) due to adverse reactions including dizziness, nausea, and joint pains. He denied any history of anabolic steroid use, long-term glucocorticoid therapy, head trauma, or systemic infections.

On arrival at the A&E, he was hypotensive with a blood pressure at 89/50 mmHg and sinus tachycardia at 120 beats per minute. Regarding general appearance, he looked very pale, acutely unwell, and lethargic, though clinically euvolemic. No palmar or buccal hyperpigmentation was observed. Neurological examination revealed no cranial nerve palsies, focal deficits, or visual field defects to suggest a compressive pituitary lesion. There was a small pinkish macular nodule measuring 1×1.5 cm on his right cheek.

Following the administration of intravenous hydrocortisone in A&E, his hemodynamics rapidly improved with blood pressure normalizing to 126/71 mmHg without a postural drop. Subsequently, his clinical appearance stabilized. He also has a slightly low sodium level on arrival (Table 1), which later returned to normal after hydrocortisone replacement in addition to withdrawal of ramipril, which he was taking for essential hypertension.

**Table 1:** Laboratory investigations upon admission Na: sodium; K: potassium; Hb: hemoglobin; WBC: white blood cell; Plt: platelets; TSH: thyroid-stimulating hormone; CRP: C-reactive protein; ALT: alanine aminotransferase

Parameter	Result	Reference range
Na	131 mmol/L	133-146 mmol/L
K	4.1 mmol/L	3.5-5.3 mmol/L
Urea	2.5 mmol/L	2.5-7.8 mmol/L
Creatinine	68 umol/L	64-104 umol/L
Blood glucose	3.8 mmol/L	>4 mmol/L
Hb	125 g/L	135-180 g/L
WBC	5.7×10⁹/L	4-11×10⁹/L
Plt	323×10⁹/L	150-400×10⁹/L
Random cortisol	<11 nmol/L	>300 nmol/L
TSH	1.6 mIU/L	0.38-5.33 mIU/L
CRP	3 mg/L	<5 mg/L
ALT	19 IU/L	0-50 IU/L

Management and diagnostic workup

Following stabilization, the case was reviewed by the endocrinology team, raising a high clinical suspicion of ICI-induced adrenal insufficiency. The patient was discharged on an oral hydrocortisone replacement regimen (10 mg in the morning, 5 mg in the afternoon, and 5 mg in the evening) and was comprehensively educated on "sick day rules", provided with a steroid alert card, and issued an emergency intramuscular hydrocortisone kit.

Outpatient diagnostic testing was conducted after holding hydrocortisone for 24 hours (Table 2). A Short Synacthen Test (SST) confirmed adrenal insufficiency with an inadequate cortisol response. Furthermore, profoundly suppressed adrenocorticotropic hormone (ACTH) and gonadotropin levels (follicle-stimulating hormone (FSH) and luteinizing hormone (LH)) confirmed a central etiology, establishing the diagnosis of secondary adrenal insufficiency due to hypopituitarism.

**Table 2:** Comprehensive outpatient pituitary hormonal profile ACTH: adrenocorticotropic hormone; IGF-1: insulin-like growth factor-1; FSH: follicle-stimulating hormone; LH: luteinizing hormone; TSH: thyroid-stimulating hormone; FT4: free thyroxine

Parameter	Result	Reference range	Comment
Baseline cortisol	26 nmol/L	>300 nmol/L	Abnormal baseline
30-min cortisol (post-Synacthen)	122 nmol/L	>450 nmol/L	Insufficient release
ACTH	<4 ng/L	9-52 ng/L	Low
IGF-1	24 nmol/L	5.4-24.5 nmol/L	Normal
FSH	0.6 IU/L	1.3-19.3 IU/L	Low
LH	<0.2 IU/L	1.24-8.62 IU/L	Low
Testosterone	<0.4 nmol/L	6.07-27.1 nmol/L	Low
Progesterone	0.4 nmol/L	0.23-4.4 nmol/L	Normal
TSH	2.4 mIU/L	0.38-5.33 mIU/L	Normal
FT4	11.5 pmol/L	8-18 pmol/L	Normal
Prolactin	232 mU/L	56-280 mU/L	Normal

A retrospective review of the patient's baseline cortisol prior to the initiation of pembrolizumab (Table 3) demonstrated normal adrenal function, establishing a clear temporal relationship between the immunotherapy and the onset of endocrinopathy.

**Table 3:** Trend of early morning cortisol levels relative to pembrolizumab administration

Date	Early morning cortisol (nmol/L)	Clinical interpretation
6 months ago	419	Sufficient (baseline)
4 months ago	240	Satisfactory
3 months ago	332	Sufficient
On the day of admission	<11	Very low, insufficient (admission)

Follow-up and outcome

Two weeks after discharge, the patient attended a dermatology-oncology joint multidisciplinary team (MDT) meeting, where he underwent a head computed tomography (CT) (Figure 1) along with a whole-body CT for restaging. Those revealed a 25 mm subcutaneous soft tissue abnormality in the right cheek, suspicious for local disease recurrence; however, there was no evidence of intracranial metastases. It makes the possibility of intracranial metastasis unlikely as a cause of his pituitary dysfunction.

**Figure 1:**
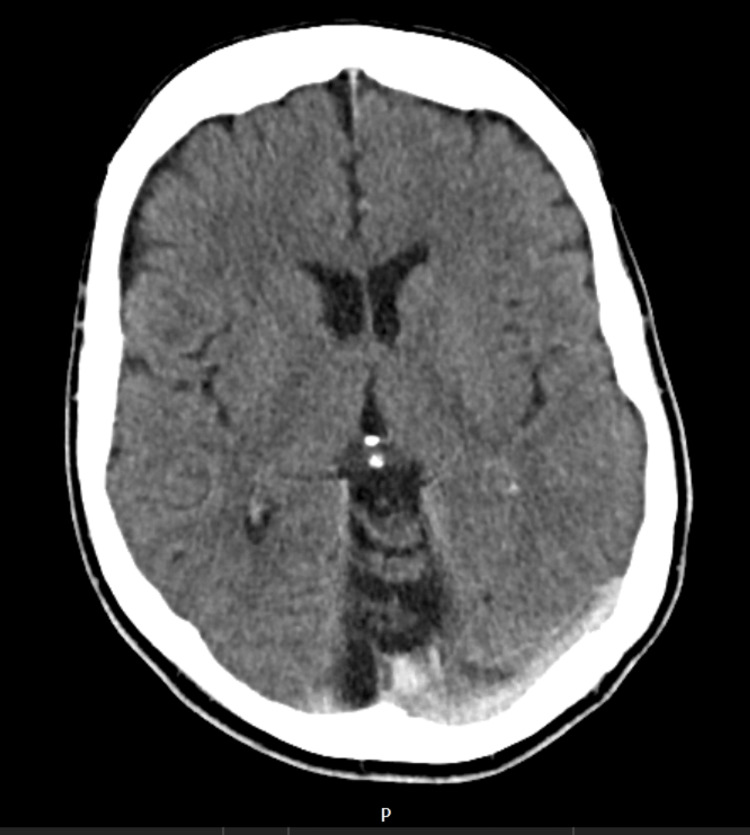
Head computed tomography showing no evidence of intracranial metastases

An outpatient magnetic resonance imaging (MRI) of the pituitary (Figure 2) was also performed after discharge to exclude pituitary adenoma or metastatic disease, and the results were normal. 

**Figure 2:**
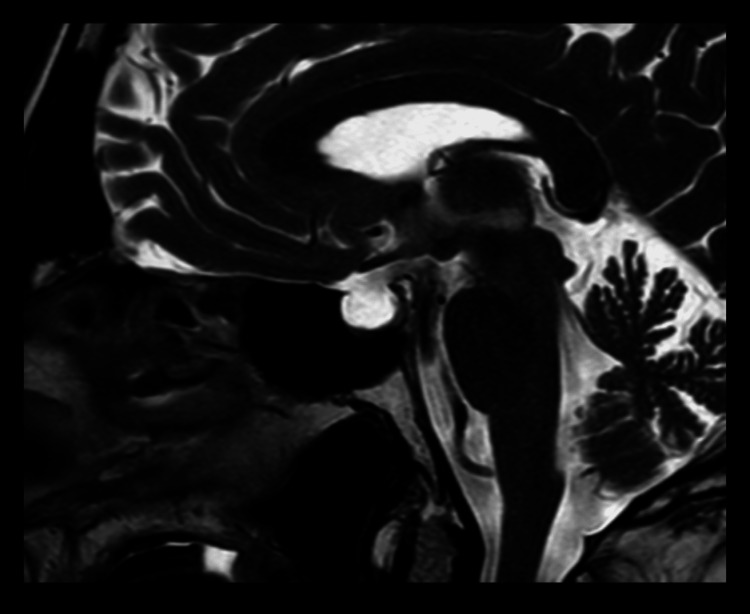
Magnetic resonance imaging of the pituitary with contrast demonstrating normal pituitary gland and stalk with no evidence of adenoma or metastatic involvement

On review by the endocrine team via telephone conversation after discharge (Table 4), it was noted that his hypopituitarism symptoms significantly improved. He was advised to continue the current regimen of hydrocortisone replacement therapy along with Nebido (testosterone) injections 1000 mg every three months, with the first two doses six weeks apart for hypogonadotropic hypogonadism. A further face-to-face endocrine clinic appointment was also made, along with dermatological and oncological face-to-face outpatient follow-ups, for the continuation of management of his superficial spreading melanoma.

**Table 4:** Summary of the clinical timeline ICI: immune checkpoint inhibitor; MDT: multidisciplinary team; CT: computed tomography

Timeframe	Clinical event/finding	Management/outcome
April 2025	Superficial spreading melanoma, right cheek	Surgical excision
August 2025	Wide local excision showed microsatellites (stage 3B)	Sentinel lymph node biopsy negative
November 2025	Initiated adjuvant pembrolizumab 400 mg	Received 3 cycles in total
February 2026	Adverse reactions to pembrolizumab	Discontinuation of immunotherapy
April 2026	Presentation with impending adrenal crisis	Intravenous hydrocortisone; oral replacement started
May 2026	Endocrine confirmation of ICI-hypopituitarism	Endocrine clinic follow-up; dose adjustment
May 2026	Dermatology/oncology MDT review	Planned CT and local sampling for melanoma recurrence
June 2026	Outpatient telephone monitoring	Symptoms well-controlled; continued steroid replacement along with testosterone replacement therapy

## Discussion

Hypopituitarism encompasses a diverse range of congenital and acquired etiologies, including pituitary adenomas, infiltrative disorders (sarcoidosis, hemochromatosis), Sheehan's syndrome, apoplexy, infectious processes, and iatrogenic causes such as cranial irradiation or exogenous steroid suppression [3]. Increasingly, ICIs, including PD-1 inhibitors, are recognized as a novel and significant iatrogenic cause of pituitary dysfunction [1,2]. The exact pathophysiological mechanism underlying PD-1 inhibitor-induced hypophysitis remains incompletely understood [4,5]. It is believed that immune checkpoint blockade disrupts self-tolerance and promotes T-cell-mediated autoimmune inflammation of the pituitary gland. This inflammatory process can lead to selective or global pituitary hormone deficiencies [4,5].

Hypophysitis is a recognized endocrine irAE associated with ICIs, although its incidence varies according to the class of agent used. The condition is more commonly reported with CTLA-4 inhibitors such as ipilimumab, with reported incidences ranging from 5% to 15%, whereas it is considerably less frequent with PD-1 inhibitors such as pembrolizumab, occurring in less than 1% of treated patients [4]. Despite its lower incidence, pembrolizumab-induced hypophysitis remains clinically important because it may present insidiously and can progress to life-threatening adrenal insufficiency if unrecognized [4].

When patients present with non-specific constitutional symptoms such as profound fatigue, anorexia, dizziness, or mood alterations, standard diagnostic algorithms often prioritize structural lesions or primary adrenal failure. Compressive symptoms like bitemporal hemianopia or early morning vomiting are typically ruled out. However, in the modern oncological era, obtaining a meticulous pharmacological history focusing on PD-1, PD-L1, or CTLA-4 inhibitors is paramount.

The diagnosis of ICI-induced hypophysitis can be challenging because symptoms such as fatigue, weakness, anorexia, weight loss, nausea, and dizziness are common among patients with advanced malignancy and may be incorrectly attributed to cancer progression, chemotherapy-related adverse effects, ageing, or general deconditioning [6]. Consequently, a high index of suspicion is required in patients receiving immunotherapy who develop new constitutional symptoms [7]. Early endocrine evaluation, including measurement of morning cortisol, ACTH, thyroid function tests, and other pituitary hormones, is essential to avoid delays in diagnosis.

In our case, the patient developed progressive symptoms of adrenal insufficiency approximately six months after the initiation of pembrolizumab therapy. He presented with fatigue, anorexia, dizziness, reduced exercise tolerance, hyponatraemia, hypotension, and profoundly low cortisol concentrations. Biochemical assessment demonstrated a failed response to the SST together with suppressed ACTH levels, confirming secondary adrenal insufficiency. Furthermore, suppressed gonadotropins and testosterone levels indicated additional anterior pituitary involvement. Importantly, baseline cortisol concentrations (Table 3) before pembrolizumab initiation were normal, supporting a causal relationship between pembrolizumab exposure and subsequent pituitary dysfunction.

A patient with pembrolizumab-induced isolated ACTH deficiency presenting with severe fatigue and hyponatraemia has previously been reported, highlighting the diagnostic challenge posed by the non-specific nature of symptoms [2]. Similar to our case, symptoms developed gradually over several months and were initially indistinguishable from cancer-related fatigue [2]. However, unlike previously reported cases with isolated ACTH deficiency, our patient demonstrated concomitant gonadotropin deficiency, suggesting broader anterior pituitary involvement while preserving thyroid and growth hormone axes.

In this case, pembrolizumab was discontinued because of adverse effects unrelated to the endocrinopathy. However, the decision to continue or temporarily withhold ICI therapy depends on the severity of the endocrine adverse event [8]. According to the European Society of Endocrinology (ESE) Clinical Practice Guideline on endocrine-related adverse conditions associated with ICIs, a well-controlled endocrinopathy is not a contraindication to the initiation or continuation of ICI therapy [8]. Patients with asymptomatic or mild to moderate endocrinopathies can generally continue treatment without interruption. In contrast, patients with severe or life-threatening endocrine toxicities should be assessed jointly by oncology and endocrinology teams to determine whether temporary interruption of pembrolizumab is warranted [8].

Pembrolizumab-induced hypophysitis can cause irreversible endocrine deficits; however, partial or complete recovery of the pituitary-adrenal axis may occur over time in a subset of patients [8]. Management hinges on acute resuscitation during crises, followed by lifelong tailored hormone replacement, continuous endocrine surveillance, and robust patient education regarding stress dosing.

## Conclusions

This case demonstrates that the presentation of vague, progressively worsening constitutional symptoms in an oncology patient should immediately prompt an investigation into therapy-induced endocrinopathies. Early endocrine assessment and a high index of suspicion are essential. This case distinctly highlights that individuals receiving pembrolizumab are at risk for life-threatening irAEs, including hypophysitis and secondary adrenal insufficiency. Missed or delayed diagnoses can precipitate fatal adrenal crises. Multidisciplinary collaboration between emergency medicine, oncology, and endocrinology is essential to ensure safe management, facilitate prompt hormone replacement, and monitor for potential future recovery of pituitary function.
